# Effect of F_1_ and F_2_ generations on genetic variability and working steps of doubled haploid production in maize

**DOI:** 10.1371/journal.pone.0224631

**Published:** 2019-11-11

**Authors:** Evellyn Giselly de Oliveira Couto, Mayara Neves Cury, Massaine Bandeira e Souza, Ítalo Stefanine Correia Granato, Miriam Suzane Vidotti, Deoclécio Domingos Garbuglio, José Crossa, Juan Burgueño, Roberto Fritsche-Neto

**Affiliations:** 1 Department of Genetics, “Luiz de Queiroz” College of Agriculture, University of São Paulo, Piracicaba, São Paulo, Brazil; 2 Instituto Agronômico do Paraná-IAPAR, Paraná, Brazil; 3 Biometrics and Statistics Unit, International Maize and Wheat Improvement Center (CIMMYT), DF, Mexico; United States Department of Agriculture, UNITED STATES

## Abstract

For doubled haploid (DH) production in maize, F_1_ generation has been the most frequently used for haploid induction due to facility in the process. However, using F_2_ generation would be a good alternative to increase genetic variability owing to the additional recombination in meiosis. Our goals were to compare the effect of F_1_ and F_2_ generations on DH production in tropical germplasm, evaluating the R*1-navajo* expression in seeds, the working steps of the methodology, and the genetic variability of the DH lines obtained. Sources germplasm in F_1_ and F_2_ generations were crossed with the tropicalized haploid inducer LI-ESALQ. After harvest, for both induction crosses were calculated the haploid induction rate (HIR), diploid seed rate (DSR), and inhibition seed rate (ISR) using the total number of seeds obtained. In order to study the effectiveness of the DH working steps in each generation, the percentage *per se* and the relative percentage were verified. In addition, SNP markers were obtained for genetic variability studies. Results showed that the values for HIR, ISR, and DSR were 1.23%, 23.48%, and 75.21% for F_1_ and 1.78%, 15.82%, and 82.38% for F_2_, respectively. The effectiveness of the DH working step showed the same percentage per se value (0.4%) for F_1_ and F_2_, while the relative percentage was 27.2% for F_1_ and 22.4% for F_2_. Estimates of population parameters in DH lines from F_1_ were higher than F_2_. Furthermore, population structure and kinship analyses showed that one additional generation was not sufficient to create new genotype subgroups. Additionally, the relative efficiency of the response to selection in the F_1_ was 31.88% higher than F_2_ due to the number of cycles that are used to obtain the DH. Our results showed that in tropical maize, the use of F_1_ generation is recommended due to a superior balance between time and genetic variability.

## Introduction

Developing doubled haploid (DH) lines in maize has become a common practice in public and private institutions worldwide because of the gain of time in plant breeding programs. The rapid development of DH lines provides more reliable selection than lines obtained through consecutive self-pollination because DH has the whole genome duplicated and thus, it means all its loci is homozygous. In summary, DH methodology includes the following steps: 1) induction of maternal haploids by crossing an inducer line with a donor genotype, 2) identification of haploids at the seed or seedling stage, 3) chromosome doubling of putative haploids selected, 4) self-pollination of the D_0_ plants to obtain D_1_ lines [1], and 5) multiplication of D_1_ lines to be introduced into the breeding program. DH production can occur in a haploid induction cross with F_1_, F_2_ or synthetic populations. Currently, breeding programs prefer to use F_1_ generation as the base population for haploid induction [2,3], while haploid inductions from F_2_ generation have been little discussed in the scientific community.

Among the advantages of using F_1_ generation in haploid induction, the possibility of maintaining favorable combinations from the parental lines and the time saved in this process can be highlighted. However, the constant use of F_1_ generation over selection cycles could result in a decreased response to selection due to a lower recombination rate in the DH lines compared to the maize lines obtained from the recombinant population [4,5]. In contrast, the use of F_2_ generation for haploid induction requires one more cycle in the breeding process, which could increase the genetic variability through the additional recombination [6]. Each generation and synthetic population used in the DH process has advantages and disadvantages in the maize breeding. Thus, the choice of them to be used in haploid induction mainly depends on the aim of the breeding program and not on the performance of the DH lines [7]. However, it is essential to discuss this trade-off between the choice of F_1_ or F_2_ generation and genetic variability in the haploid induction approach, especially in tropical maize. At present, studies reporting this question are related to temperate maize germplasm or computational simulations [6,7].

After the crosses between haploid inducer and the source population, the next step is the identification of haploid seeds or seedlings. There are different methodologies to separate haploid from diploid in maize such as R*1-navajo* (R*1-nj*) marker [8], oil content of seeds [9], flow cytometry [10], differences in early seedling traits[11], red root marker[12], and stomata length [13,14]. Usually, the haploids seeds are selected based on anthocyanin pigmentation in the embryo controlled by the R*1-nj* because this methodology is easy, cheap, free, and seeds are classified before the artificial doubling. This phenotypic marker, however, has variable expression depending on the source germplasm used as a donor [15], mainly in the cases of inhibitor genes present in the tropical germplasm [16]. Consequently, false positives are commonly found in the haploid samples selected by R*1-nj*. In tropical maize, the choice of generation influences R*1-nj* expression. When F_1_ or F_2_ populations used for haploid induction have an inhibitor gene in their genome, kernels will segregate for the R*1-nj* phenotype. In turn, haploid kernels may not be efficiently identified, and a half to three-fourths of the haploids could potentially be lost [17]. In this case, donor genotype that has inhibitor genes in their genome can have limited use in DH technology. Chaikan et al. [16] analyzed the effectiveness of R*1-nj* anthocyanin in haploid induction from different tropical lines and showed that anthocyanin phenotype could be completely suppressed or poorly expressed in some germplasm, making it impossible or inefficient to identify haploids at the seed stage. Although tropical source germplasm influences on the expression of R*1-nj* anthocyanin in induced seeds, the effects of inhibitor genes in the induction of F_1_ and F_2_ (F_1_/F_2_) generations of this genetic background has not yet been studied, especially considering the commercial genotypes. Moreover, a comparison of maize haploid inductions in F_1_/F_2_ generations for DH working steps and effectiveness by step has not been studied in detail. This knowledge could help breeders to identify the critical steps in DH production and drive improvement of tropical haploid inducers, as well as direct the logistics planning necessary for each phase of the methodology.

In this context, the goal was to compare the effect of F_1_/F_2_ generations on DH production in tropical germplasm, evaluating (i) the R*1-nj* expression in seeds, (ii) the practical steps used in the methodology, and (iii) the genetic variability estimates of the DH lines obtained.

## Materials and methods

### Plant material

Five commercial single-cross hybrids were selected to represent Brazilian germplasm marketed by private companies (Table 1). Currently, the maize crop in Brazil is represented mostly by hybrid cultivars (88.32%) [18]. In order to study different generations in DH methodology, F_1_ hybrids were selfed to produce F_2_ populations in the summer cycle of October 2013 to February 2014 at Luiz de Queiroz College of Agriculture–ESALQ/USP, Piracicaba, Brazil.

**Table 1:** Source germplasm used in maize haploid induction.

Source germplasm	Grain texture	Cycle	Transgenic	Company
2B587PW	Semident	Premature	Yes	Dow
30F53H	Softflint	Premature	Yes	Pioneer
DKB390	Softflint	Premature	No	Dekalb
STATUS VIPTERA	Flint	Premature	Yes	Syngenta
BM820	Flint	Premature	No	Biomatrix

### Haploid induction in a tropical climate

In order to obtain haploid seeds, induction crosses were performed using the tropical inducer LI-ESALQ as pollinator of F_1_/F_2_ generations from different source germplasm. This inducer is derived from a cross of two inducer lines (W23 and Stock6) with a maize hybrid adapted to tropical conditions, and it has *R1-nj* marker responsible for anthocyanin expression in the endosperm and embryo [8].

Induction crosses occurred in the summer cycle of October 2014 to February 2015 at the University of São Paulo/ESALQ in Piracicaba, Brazil. Aiming prevents contamination with other pollens, induction crosses were developed in an isolated field area. A randomized complete block design was used with three replicates. Seeds were planted in 7.0 m rows with a spacing of 0.85 m between rows. To provide ideal conditions for pollination, tropical inducer LI-ESALQ was planted in rows interspersed with source germplasm rows on three different days: on the same day that germplasm sources were planted and five and ten days later. At flowering, female lines were detasseled every day, to the begging until the last flowering day, to enable natural pollination by the inducer.

### Seed selection based on anthocyanin expression

Based on the expression of *R1-nj* marker [8], seeds were separated and grouped into three categories: 1) putative haploids: seeds with a white embryo and purple endosperm; 2) diploids: seeds with purple embryo and endosperm; and 3) inhibitors: seeds with a total absence of purple coloring.

A total of kernels selected as putative haploids, diploids, and inhibitors based on this morphological marker were used in statistical analyses.

### Germination and artificial chromosome doubling

After separating seeds, putative haploids were germinated and kept at a controlled temperature of 25°C for 72 hours. Vigorous seedlings (a typical diploid phenotype) were considered false positives and discarded [19].

For artificial chromosome doubling, seedlings were treated with 0.06% colchicine and 0.75% dimethyl sulfoxide (DMSO) solution for 12 hours [20] (in this study we modified the percentage of DMSO used), and it was kept in the dark at ambient temperature (mean of 23.7°C). After the 12 hours of the doubling process, seedlings were rinsed in water for 40 minutes and then transferred to plastic cups containing substrate. Seedlings were irrigated twice a day and kept for seven days in a greenhouse located at the Genetics Department of ESALQ/USP.

Effectiveness of the DH working steps was verified by the overall number of germinated and duplicated seeds and the number of surviving seedlings.

### Field experiment after chromosome doubling

After the chromosome doubling and the time of seven days in the greenhouse, young plants were transplanted to the field at the experimental area of the Genetics Department of ESALQ/USP. It was not used any experimental design in this step. One month after transplanting, false positives were discarded based on their phenotype. Haploid plants are considered less vigorous, and with narrower and more erect leaves than hybrids [21]. Thus, vigorous plants, with a thick, anthocyanin colored stalk, and highly branched tassel were removed. Subsequently, only D_0_ plants remained in the field, which allowed the estimation of false discovery rate.

The false discovery rate (FDR) refers to the probability of a sample being genuinely harmful. In other words, it is the proportion of diploid plants present in the group selected as haploid, which was estimated by the following equation, according to Melchinger et al. [9]:

$$\text{FDR}=\frac{\text{FP}}{\text{TP}+\text{FP}}$$

Where, in this study, FP (false positive) was the number of diploid plants in the field after the roguing and TP (true positive) was the number of haploid plants that remained in the field.

At the flowering stage, D_0_ plants were artificially self-pollinated to obtain D_1_ lines. In this step, the total number of D_0_ plants and the number of D_0_ plants that were self-pollinated were counted.

Finally, at the end of D_0_ plants cycle, ears were harvested and selected according to size, number of seeds, and R*1-nj* expression.

### Analyses of phenotypic data

The data obtained from seed selection in the categories of putative haploids, diploids, and inhibitors were used for statistical analyses. After, we estimated haploid induction rate (HIR), inhibition seed rate (ISR), and diploid seed rate (DSR). In addition, data obtained in this study were categorical for independent variables, which consisted of the counts obtained in each seed category (HIR, ISR, and DSR). Therefore, a generalized linear mixed model with multinomial logit distribution was used. Diploid was the reference category. This model allowed to predict the probabilities of different seed rates for germplasm sources and generations used:

$$Y_{ktj}=\mu +S_{k}+G_{t}+{\left(SG\right)}_{kt}+B_{j}+\varepsilon_{ktj}$$

*Y* ~ Multinomial (*N*, *π*), $B_{j}\sim \text{N}(0,\sigma_{B}^{2})$, *ε*_*ktj*_ ~ N(0, *σ*^2^), in which *Y*_*ktj*_ is the value of HIR, ISR, and DSR in source germplasm *k*, generation *t* and block *j*, *μ* is the overall mean value, *S*_*k*_ is the fixed effect of source germplasm *k*, *G*_*t*_ is the fixed effect of generation *t*, *(SG)*_*kt*_ is the fixed effect of germplasm source × generation interaction, *B*_*j*_ is the random effect of block *j*, and *ε*_*ktj*_ is the random effect of experimental error.

The binomial distribution used in *logit* function is expressed by:

$$\begin{array}{cc} g(.)=\text{ln}(\frac{\pi_{kti}}{1-\pi_{kti}}) & \pi_{i}=\frac{e^{X_{i}^{\prime }\beta }}{1+e^{X_{i}^{\prime }\beta }} \end{array}$$

where *π*_*kti*_ is the probability of haploid, diploid, or inhibited seeds in generations *t* and source germplasm *k* in the i-eth observation unit (total amount of seeds).

These analyses were carried out using PROC GLIMMIX procedure of SAS software (SAS UNIVERSITY EDITION, 2018). Mean values of HIR, ISR, and DSR were discriminated for generation and genotype within each generation, by t-test with R graphics package in software R 3.5.0 (R Development Core Team, 2018). The phenotypic information used in this study can be found in (*doi*:10.17632/98t8nxgw5s.2*)*.

### Effectiveness of the working steps in obtaining doubled haploid lines

Each working step of DH obtention was used to analyze the effectiveness of this methodology, for which it was considered the percentage *per se* and relative percentage (Table 2).

**Table 2:** Working steps used to obtain DH lines in maize.

Working step	Description
1.	Total of putative haploids
2.	Chromosome doubling
3.	Greenhouse
4.	Total in the field
5.	Total in the field after roguing false positives
6.	Self-pollination of D_0_ plants
7.	Total harvested ears
8.	Total harvested D_1_ ears

The number of units present (seeds, seedlings, and plants, as well as D_1_ seeds) in each working step were used as proposed by Melchinger et al. [22]. Thus, the effectiveness of each pratical step was obtained by:

$\%=\frac{E_{n}}{E_{1}}$ percentage *per se* of each working step (*E*_*n*_) per the initial number of putative haploids (*E*_*1*_).
${\%}_{\text{R}}=\frac{E_{n}}{E_{n-1}}$ relative percentage of each working step (*E*_*n*_) per the previous step (*E*_*n-1*_).

Percentages *per se* (%) refer to the steps after germination of putative haploids. In contrast, relative percentages (%_R_) correspond to the percentage of biological material from one step per the previous step. At this step of the study, no experimental design was used; hence, the F_1_ and F_2_ generations were qualitatively compared by the (%) and (%_R_) calculations.

### Genotyping and quality control

Only the leaf samples of D_0_ plants obtained from F_1_/F_2_ generations were collected to study the genetic variability through molecular markers because the number of D_1_ lines obtained was not sufficient for genotypic analyses. A total of 95 lines in the F_1_ generation and 78 in F_2_ generation from the five commercial genotypes were used. Samples were collected after the flowering stage. Fertility is a prime indicator of DH plants [23], while haploid plants remain sterile. D_0_ plants were randomly chosen to represent all individuals in the population. Thereby, the self-pollinated plants that did not present symptoms of diseases were selected.

Samples were genotyped with 7,430 Single Nucleotide Polymorphisms (SNPs) markers. This step was carried out by DuPont-Pioneer through Illumina *GoldenGate* platform. Genotypic data were optimized for genetic variability studies, population structure, and kinship analyses. Markers that had more than 5% missing data or less than 5% minor allele frequency (MAF) [7] were excluded. Additionally, all heterozygous loci that remained in the data were considered as missing values. Then, all the missing values in the genotypic matrix were imputed [24]. The residual heterozygosity was considered as a missing value because of the presence of chimerism in D_0_ cells after artificial chromosome doubling during DH in maize [13,14].

Quality control, conversion of SNP markers into numerical algorithms and imputation of missing values were carried out by the raw.data function of *snp*Ready package [24] of R software 3.5.0 (R Development Core Team, 2018). The genotypic data file used in this study is available in (*doi*:10.17632/98t8nxgw5s.2*)*.

### Analyses of genome variation

Our aim in these analyses was to verify if the additional recombination of F_2_ generation in haploid induction could modify the variability to allow the formation of new groups. Population parameters of DH lines derived from F_1_/F_2_ generations were estimated for each SNP by group (generations) and subgroup (source germplasm) through the popgen function of the *snp*Ready package of R software 3.5.0 (R Development Core Team, 2018), namely:

1. Minor Allele Frequency, ($\text{MAF}=\frac{H_{e}+R}{2(D+H_{e}+R)})$, where *H*_*e*_ is the total heterozygous loci for SNP evaluated, D is the total homozygous loci for allele *i*, R is the total homozygous loci for allele *j*, and this last allele is of minor frequency.
2. Polymorphism Information Content, $\text{PIC}=1-\sum_{i=1}^{k}p_{i}^{2}-\sum_{i=1}^{k-1}\sum_{j=i+1}^{k}2p_{i}^{2}p_{j}^{2}$, where *p*_*i*_ and *p*_*j*_ are the frequency of *ith* and *jth* allele for SNP evaluated.
3. Nei’s gene diversity [25], $\left(D_{nn\prime }=H_{nn\prime }-\frac{H_{n}+H_{n\prime }}{2}\right)$, where D_*nn′*_ measures diversity between the n-th and the n′-th subpopulation, H_n_ is the estimate of heterozygosity in the n-th locus, and H_n’_ is the heterozygosity in the n′-th locus.
4. Estimation of the potential genetic variance (E_VG_), calculated in this study by the sum of the additive and dominance variance portions due to the allele frequencies, with V_G_ = 2pq + 4p^2^q^2^. These measures are being presented as a proxy for genetic variance since the additive, and dominant effects of the loci were not used.
5. Inbreeding effective population size, $\left(N_{e}=\sum_{i}\frac{1}{1+f_{i}}\right)$ where *f*_i_ is the individual inbreeding coefficient, which was estimated by the diagonal (*diag*(*K*) − 1), being K the kinship matrix of the individuals that compose the subpopulation.
6. Response to selection (RS = *i* r E_VG_), where *i* is the selection intensity fixed at 0.1, r is the selective accuracy at 0.5, and E_VG_ is the estimation of the potential genetic variance. Later, the estimates obtained were used to quantify the relative efficiency of the response to selection (E_RS_), by the equation ${\text{E}}_{\text{RS}\%}=(\frac{RS_{F_{1}}xT_{F_{2}}}{RS_{F_{2}}xT_{F_{1}}})x100$, where *RS* is the response to selection of F_1_/F_2_ generations and T is the number of cycles used to obtain DH lines. In terms of T value, four cycles were considered in the F_1_ generation, and five cycles in the F_2_ generation.

### Population structure and relationship

In order to study the performance of doubled haploids obtained from F_1_ and F_2_ generations, due to the additional recombination in F_2_, it was performed a population structure and kinship analyses. Principal component analysis (PCA) was carried out to study population structure through the pcaMethods R package [26]. Moreover, to evaluate the relationship among the DH lines, additive genomic kinship matrix was constructed by the method of Yang et al. [27] through *snp*Ready R package [24] of R software 3.5.0 (R Development Core Team, 2018).

## Results

### Haploid induction and *R1-navajo* marker expression

In the multinomial analysis of HIR, DSR, and ISR, significant differences (*р* < 0.05) were observed among source germplasm, generation, and source germplasm × generation interaction. A total of 415,979 seeds were obtained, where 1.51% were selected as putative haploids, 78.82% selected as diploid seeds, and 19.65% showed inhibited marker expression (Table 3). HIR of source germplasm ranged from 0.77% to 3.76%, and the highest values were observed for genotype 30F53H in both generations. ISR ranged from 4.60% to 57.75%, and the genotype 2B587PW had the highest inhibition in the generation studied. DSR ranged from 41.47% to 93.88%, and the genotype 2B587PW had the lowest rates, while genotypes 30F53H, STATUS VIPTERA, and DKB390 had the highest rates.

**Table 3:** Number of total seeds (T_S_), diploid (T_D_), haploid (T_H_), and inhibited (T_I_) seeds from source germplasm and generation (F_n_) used in multinomial analyses. Means values of haploid induction rate (HIR), inhibition seed rate (ISR), and diploid seed rate (DSR) are also presented.

Source germplasm	F_n_	T_D_	T_I_	T_H_	T_S_	HIR %		ISR %	DSR%
						Mean	Range	Mean	Mean
30F53H	F_1_	53293	6371	1598	61262	2.60^a^	0.17–8.01	10.40^c^	87.00^c^
2B587PW		20359	28408	383	49150	0.77^c^	0.14–4.78	57.75^a^	41.47^e^
STATUS VIPTERA		56289	5603	614	62506	0.97^b^	0.18–5.15	8.94^c^	90.08^b^
BM820		32504	17196	402	50102	0.80^bc^	0.13–4.46	34.44^b^	64.76^d^
DKB390		51546	3292	582	55420	1.05^b^	0.17–5.88	5.91^d^	93.03^a^
Total		213991	60870	3579	278440	-	-	-	-
Mean		-	-	-	-	1.23^B^	-	23.48^A^	75.21^B^
30F53H	F_2_	26276	1651	1054	28981	3.76^a^	0.20–15.49	5.48^c^	90.75^a^
2B587PW		12958	9774	186	22918	0.85^c^	0.17–4.79	41.75^a^	57.39^c^
STATUS VIPTERA		35659	1762	569	37990	1.51^b^	0.21–7.59	4.60^c^	93.88^a^
BM820		15085	4370	225	19680	1.25^bc^	0.19–8.05	21.12^b^	77.63^b^
DKB390		25811	1724	435	27970	1.53^b^	0.21–3.96	6.19^c^	92.27^a^
Total		115789	19281	2469	137539	-	-	-	-
Mean		-	-	-	-	1.78^A^	-	15.82^B^	82.38^A^
Total		329780	80151	6048	415979	-	-	-	-
Overall Mean		-	-	-	-	1.51	-	19.65	78.82

^†^Means followed by the same letter (uppercase compare genotype mean within each generation; lowercase compare mean of generations) in the column are not significantly different at the 0.05 probability level.

**Fig 1:**
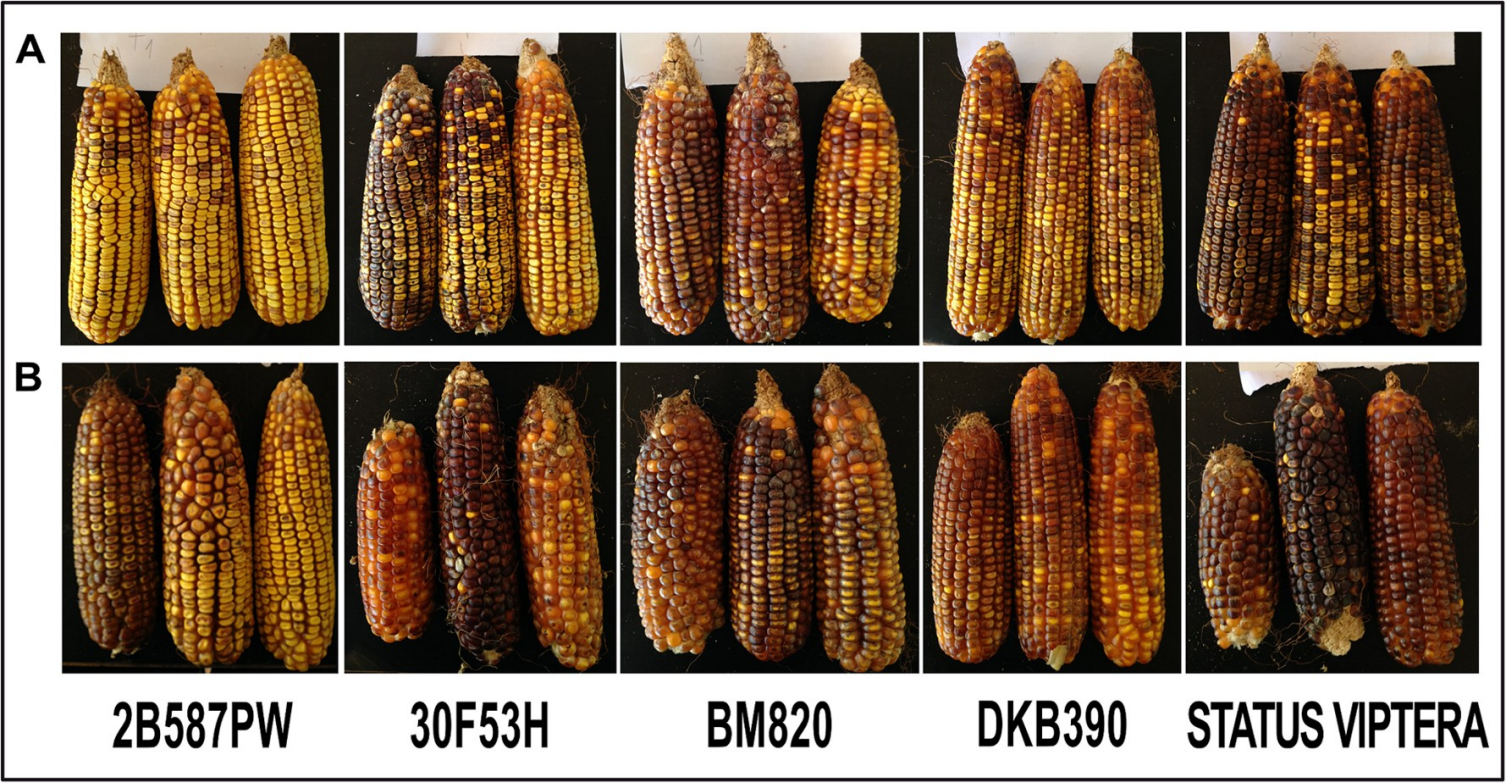
Variation in the intensity of *R1-nj* expression in seeds. A. Generation F_1_; B. Generation F_2_.

The F_2_ generation showed higher HIR (1.78%) and DSR (82.38%) than F_1_ generation (1.23% and 75.21%, respectively). A lower ISR was observed in the F_2_ generation (15.82%) than in F_1_ generation (23.48%) (Table 3). Moreover, *R1-nj* expression was more evident in F_2_ than in F_1_, indicating that the inhibitory genes present in the commercial hybrids were heterozygous [16]. Thus, with the additional recombination in the F_2_ generation, heterozygous genes segregated and enabled R*1-nj* expression in the seeds (Fig 1). Comparing the source germplasm used in this work, ears of genotype 2B587PW had the least purplish color, consistent with the results for ISR.

Analyses of the false discovery rate (FDR) showed mean values of 52.12% (Table 4). Source germplasm varied from 63.82% to 21.59% in the F_1_ generation and 66.77% to 40% in F_2_.

**Table 4:** False discovery rate (FDR) in source germplasm and F_1_/F_2_ generations.

Source germplasm	Generation	FDR %
30F53H	F_1_	63.82
	F_2_	66.77
2B587PW	F_1_	55.10
	F_2_	64.84
STATUS VIPTERA	F_1_	44.16
	F_2_	40.00
BM820	F_1_	21.59
	F_2_	43.52
DKB390	F_1_	49.77
	F_2_	49.09
Overall mean		52.12

### Percentage *per se* and relative percentage on working steps

In the effectiveness of the working steps the initial number of seeds varied among each source germplasm and generation, and all of them were used in this study. So, after germination of putative haploids and discard of vigorous seedlings, 46.4% of total seedlings were used in chromosome doubling in both generations (Table 5). Percentage *per se* of plants in the greenhouse was 36.6%. In the field, the percentage *per se* was 31.1% before roguing and 14.8% after roguing. D_0_ flowering occurred about 45 days after germination. In this step, all the plants that had a fertile tassel and compatible stigma were self-pollinated (5.9%). At the end of the maize cycle, ears harvested showed a percentage *per se* of 1.6% and ears selected as D_1_ lines showed a percentage *per se* of 0.4%. In summary, from 6048 putative haploid seeds, 27 D_1_ lines were obtained when LI-ESALQ inducer was used.

**Table 5:** Working steps used to obtain doubled haploid lines in tropical maize considering total and individual values in the F_1_/F_2_ generations. % is the percentage *per se* and % _R_ is the relative percentage.

Working steps				F_1_			F_2_		
	Total	%	% _R_	Total	%	% _R_	Total	%	%_R_
0. Total seeds				278440			137539		
1. Total putative haploids	6048	100	-	3579	100	1.2	2469	100	1.7
2.Chromosome doubling	2868	47.4	47.4	1681	47.0	47.0	1187	48.0	48.0
3.Greenhouse	2218	36.6	77.3	1362	38.0	81.0	856	34.6	72.1
4.Total in the field	1882	31.1	84.8	1170	32.6	85.9	712	28.8	83.1
5.Total in the field after roguing false positives	901	14.8	47.8	585	16.3	50.0	316	12.7	44.3
6.Self-pollination of D_0_ plants	358	5.9	39.7	222	6.2	37.9	136	5.5	43.0
7.Total harvested ears	99	1.6	27.6	50	1.3	22.5	49	1.9	36.0
8.Total harvested D_1_ ears	27	0.4	27.2	16	0.4	32.0	11	0.4	22.4

Percentage *per se* of false positives in the field was 47.8% after rouging. It might happen due to the inducer characteristics, such as low HIR and a high proportion of false positives due to *R1-nj* expression. For each generation, more biological material was observed in F_1_ generation than in F_2_ generation. However, the amount of biological material did not affect DH methodology.

Chromosome doubling step had about the same percentage *per se* in F_1_/F_2_ (47% and 48%, respectively). After this step, until the self-pollination of D_0_ plants, F_1_ generation presented higher values than the F_2_ generation. Harvested ears step showed higher values of percentage *per se* and relative percentage in F_2_. Even F_1_/F_2_ generations had shown variation across the working steps, the rate of D_1_ lines obtained was the same (0.4%), indicating that generation did not affect the portion of DH lines obtained (Table 5).

Percentage *per se* in the working steps varies among source germplasm, indicating that the success of the methodology depends on the genetic background.

### Quality control in the SNP marker data

A total of 7,430 SNPs markers were used in the DH lines genotyping. However, about 1826 markers were eliminated by quality control. Hence, 173 individuals and 5604 markers were used in the analyses of genetic variability and population structure.

### Genetic variability and response to selection

Analyses of genetic variability were performed at the F_1_/F_2_ generation and at the subgroup (source germplasm) (Table 6, S1 Table). Inbreeding effective population size (N_e_) was higher in DH lines derived from F_1_ (47.56) as also the estimation of the potential genetic variance (E_VG_) (2809.67). Mean values of genetic diversity (D_G_), polymorphic information content (PIC), minor allele frequency (MAF), and response to selection (RS) were also higher among DH lines derived from F_1_ generation than those derived from F_2_. Finally, the relative efficiency of the response to selection (E_RS%_) in DH lines of the F_1_ generation was 31.88% higher than that of F_2_.

**Table 6:** Population parameters estimates of DH lines obtained from five source germplasm and generations (F_1_ and F_2_). Number of individuals (N°), inbreeding effective population size (N_e_), estimation of the potential genetic variance (E_VG_), Nei’s genetic diversity (D_G_), polymorphic information content (PIC), minor allele frequency (MAF), coefficient of inbreeding (F_i_), and response to selection (RS). In parentheses are the maximum and minimum values.

Generation	N°	N_e_	E_VG_	D_G_	PIC	MAF	F_i_	RS
F_1_	95	47.56	2809.67	0.36 (0.04–0.50)	0.29(0.03–0.38)	0.27 (0.02–0.50)	1.00 (0.99–1.00)	140.48
F_2_	78	39.10	2663.1	0.34 (0–0.50)	0.27 (0–0.38)	0.25 (0–0.50)	1.00 (0.99–1.00)	133.15

### Population structure and genetic relationship

**Fig 2:**
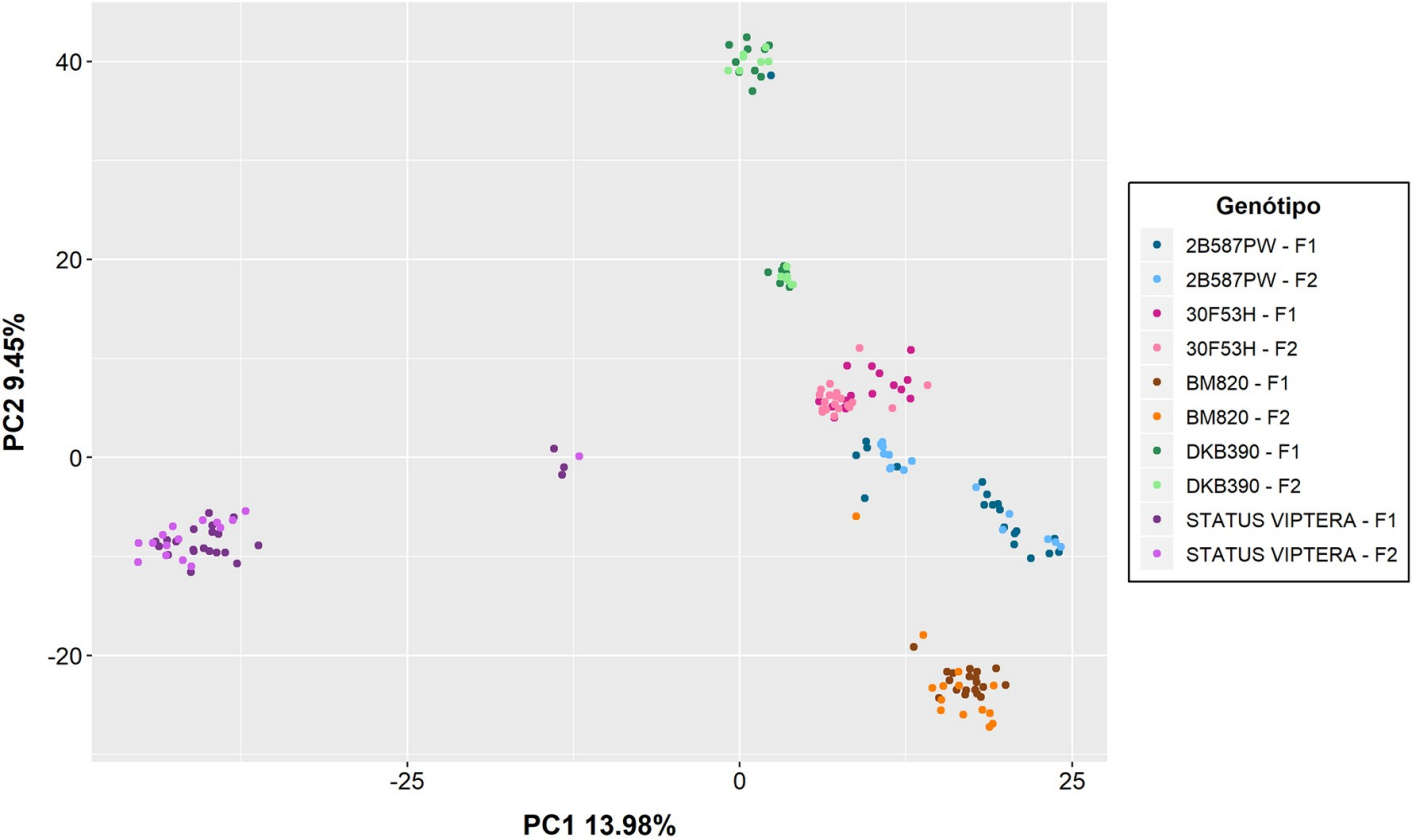
Principal Component Analysis (PCA) in DH lines obtained from five source germplasm and F_1_/F_2_ generations.

PCA analysis grouped source germplasm into four groups. The first with the genotype DKB390, the second with the genotypes 30F53H and DKB390, the third with the genotype STATUS VIPTERA, and the fourth the genotype BM820 (Fig 2). There was no separation of subgroups due to the F_1_ and F_2_ generations within each source germplasm, indicating that additional recombination in the DH lines from F_2_ generation was not sufficient to create new subgroups.

**Fig 3:**
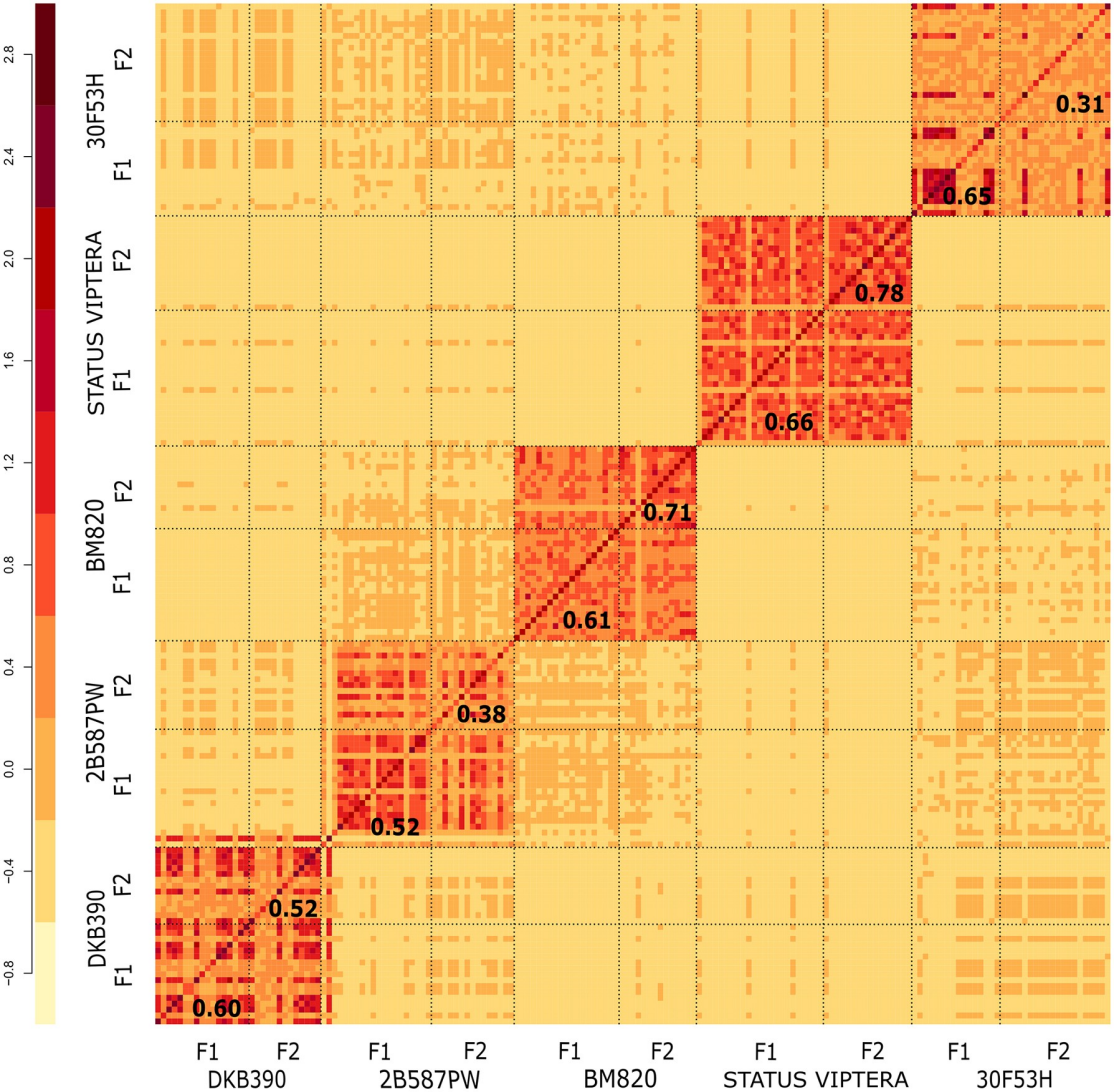
Heatmap of the kinship coefficient of 173 DH lines obtained from five source germplasm and F_1_/F_2_ generations. Number in bold within each subpopulation represents the overall kinship mean.

The five source germplasm and their F_1_/F_2_ generations were also clustered based on the genomic kinship matrix (Fig 3). The results were consistent with PCA analysis, and also indicated that one additional recombination in the F_2_ generation was not sufficient to separate subgroups within a population.

## Discussion

Inhibition genes present in tropical germplasm can difficult haploid selection in maize DH production once anthocyanin inhibition can be total or partial, depending on the alleles form in the source population used as a donor [14,25]. These mutant genes, known as C*1*-*I*, C2-I*df*, and *in*-1D, act on the anthocyanin pathway preventing its expression in seeds [28,29]. When dominant inhibitors are present, such as C*1*-*I*, inhibition in seeds is total, and selection of haploids by seed color is not possible [16]. When R*1-nj* locus segregates, it is possible to identify some haploids, but not all of them, due to the absence of purple coloration in the endosperm. In this study, inhibition genes acted in R*1-nj* expression because HIR, ISR, and DSR varied among source germplasm and generations (Fig 1 and Table 3). F_2_ generation showed higher HIR, DSR, and lower ISR than F_1_, which means that inhibition alleles underwent additional recombination in the F_2_ generation, causing segregation in these alleles. In Fig 1, it is possible to compare anthocyanin variation among and within ears from F_1_ and F_2_ generation.

FDR analyses showed that the number of seeds selected by the *R1-nj* marker does not mean more success in the selection of true haploids, due the presence of false positives in the number of seeds selected as haploid (Table 4). Genotype 30F53H showed higher HIR in both generations, but its FDR was 65.29%, indicating that the selection of many putative haploid seeds by R*1-nj* does not indicate the selection of more haploid seeds. In contrast, genotype 2B587PW had the highest ISR, indicating that its genetic background inhibits anthocyanin expression in a higher percentage of the seeds. These results show the importance of the choice of generation and source germplasm to be used in haploid induction in tropical maize as well as the haploid inducer line. The LI-ESALQ inducer line used in this work had an FDR that ranged from 21.59% to 66.77% (Table 4). Misclassification rates associated with the *R1-nj* can be quite substantial (≥30%) depending on the source germplasm used in the induction crosses [12,15], while the haploid rate depends exclusively on the haploid inducer. This means that, when a haploid inducer has a higher HIR, as temperate inducer lines with 8–15% [11,15], the number of haploid seeds will be higher in the amount of seeds obtained in the induction crosses. In other words, a minor number of seeds induced would be used to perform the DH methodology. In our study, the overall mean of HIR was 1.5%, which is lower than temperate inducers. However, we used an amount of 329,780 seeds to obtain a number of haploid sufficient to conduct the experiment (Table 3). Improving the success of HIR in LI-ESALQ inducer line could be an alternative to reduce the cost and time with induction crosses and seed selection. In this sense, a specific breeding program can increase the HIR in this genotype, because the artificial selection could pressure in the *sed 1* locus responsible for the haploid induction [30].

For HIR, ISR, and DSR values, the best generation to induce haploids in tropical maize should be F_2_, given that the segregation of inhibitory alleles would enable greater haploid selection and lower loss of inhibited seeds. However, selecting F_2_ generation only for this purpose may not be efficient, since the time necessary to obtain DH lines needs of one cycle more. In addition, the HIR difference between F_1_ (1.23%) and F_2_ (1.78%) was 0.55%, that is, the low value does not justify the time and resources of an additional cycle for haploid induction from F_2_. Furthermore, the F_2_ generation also exhibited lower values of N_e_, E_VG_, D_G_, MAF, PIC, and RS than the F_1_ (Table 6). Due to an additional recombination in F_2_, it was expected that DH populations from F_2_ had higher population parameters estimates than F_1_, which was not observed. DH lines derived from different germplasm sources showed delimitated groups between populations in the kinship analysis, which represent the different maize germplasms of private companies used in this work. Even in population structure and kinship analyses, results showed that additional recombination in the F_2_ generation were not sufficient to create genetic variability in DH lines. Moreover, the E_RS%_ was 31.88% greater in DH lines of F_1_ generation due to the shorter time used when compared with the F_2_ generation. According to Sleper et al. [7], the decision of inducing haploids in F_1_ or F_2_ generation needs to consider factors other than the performance of the resulting DH lines. Therefore, F_1_/F_2_ generation and the amount of biological material did not affect the efficiency in obtaining DH lines (Table 5). The working steps approach can help the breeder to optimize the number of seeds in the induction, the space in the field and greenhouse, and money needed for the laboratory activities. The loss of biological material observed in the methodology occurred because of the number of false positives and some stress factors. Colchicine duplication was the initial factor, followed by transferring the seedlings to the field and finally the rouging of false positives. The overall mean of FDR (52.12%) represented the percentage mean of false positives rouging in the field (47.8% of F_1_ and 49.9% of F_2_). In addition, high temperatures and rains during flowering reduced D_0_ fertilization, which indicates the importance of the environmental choice. Moreover, source germplasm interfered in DH methodology due to the number of false positives and sensibility to colchicine and environment factors.

Results presented in this study about working steps in DH production enables maize breeders to estimate seed quantities in the first step (induction crossing) and plan field areas that will be used. Considering the results showed in each working step, we present below some estimates related to the number of seeds that should be induced with the tropical inducer line LI-ESALQ to obtain 100 DH. The total number of DH lines obtained in each generation was used to perform the estimation: 16 in the F_1_ generation and 11 in the F_2_ generation. In order to obtain 100 DH from F_1_ generation, maize breeders should have approximately 22,368 putative haploid seeds. It would be selected by R*1-nj* marker from approximately 1,700 million of induced seeds. Thus, considering an average of 520 grains per ear, the field area necessary to perform the haploid induction crosses would be from 3,345 donor plants. Conversely, to obtain 100 DH from F_2_ generation, maize breeders should have approximately 22,445 putative haploid seeds, which would be selected from approximately 1,200 million induced seeds. Here, considering an average of 372 grains per ear, the field area necessary to perform the haploid induction crosses would be from 3,362 donor plants. In order to compare F_1_/F_2_ generation, results presented above showed that the number of putative haploid seeds (22,368 and 22,445, respectively) and the number of donor plants in the field (3,345 and 3,362, respectively) are not very divergent to justify the use of F_2_ over F_1_. Haploid induction in generation F_2_ requires less biological material than F_1_ (approximately 500,000 induced seeds). However, one additional cycle is also required. Working steps numbers showed in this study were obtained considering one environment. Considering the DH methodology and maize plant breeding, different environments can be introduced in future studies about DH lines performance or the development of new inducers lines.

The results of our study showed that the induction of haploids must continue in the F_1_ generation, while F_2_ should be used in specific objectives of the breeding program. For example, if a specific maize hybrid that has a favorable genetic diversity to be used in a doubled haploid program and shows high ISR after haploid induction crosses, the use of its F_2_ generation could be a good choice. Another advantage of the use of F_2_ generation is the possibility to select among segregating plants before haploid induction. However, the continued use of F_1_ generation in haploid induction is recommended because it avoids the laborious process of one more self-pollinating to obtain F_2_ donor plants, and it offers advantages such as saving time and resources. Additionally, nearly 90% of the donor population genome can be inherited by the haploid individuals, enabling the use of the parent’s potential in the next breeding cycles [7]. In some countries, such as Brazil, commercial hybrids can be used as donor sources, which facilitates access to the elite germplasm already present in the hybrid seed market [3]. One limitation showed in this study is the small number of the population used in the DH production. However, the commercial Brazilian maize germplasms have satisfactory genetic diversity, with the most substantial variability between companies [18], and we used five different companies’ seeds. Mainly for public institutions or small breeding programs that do not have source germplasm to start a DH methodology, haploid induction in F_1_ hybrids is an alternative for accelerating research and obtaining lines. Insertion of exotic germplasm in the breeding program can be expensive and slow, depending on the seeds importation laws of the country. Private seeds companies that already have established heterotic groups, haploid induction in F_1_ generation allows that new inbred lines are obtained with 90% of the genome preserved. In this sense, seed companies can obtain hybrids more productively than those in the commercial market. However, the use of commercial hybrids for inducing haploids should not detract from or infringe laws that protect cultivars in the countries in which they are used [3]. Besides, in some countries, the commercialization of transgenic maize seeds is allowed, which means that haploid induction from transgenic hybrids can produce DH transgenic lines. In this situation, it is essential to understand the laws and royalties to need pay to owners.

## Conclusions

The present work showed that the doubled recombination in F_2_ DH lines was not sufficient to create new groups in population structure and kinship analyses, or increase the population parameter estimates when compared with F_1_. Further, the effectiveness of the working steps analyses, F_1_/F_2_ generation showed the same percentage (%) in the total of D_1_ ears harvested, indicating that one more generation did not affect the number of DH lines obtained. Thereby, we recommended the use of F_1_ generation in doubled haploid production from tropical sources germplasm due to balance in time and genetic variability.

## Supporting information

S1 Table Estimation of population parameters of DH lines obtained from each evaluated population and generation. Generation (F_n_), number of individuals (N°), inbreeding effective population size (N_e_), estimation of the potential genetic variance (E_VG_), Nei’s genetic diversity (D_G_), polymorphic information content (PIC), minor allele frequency (MAF); coefficient of inbreeding (F_i_). In parentheses are the maximum and minimum values.
(DOCX)

## Abbreviations

D_G_: Nei’s gene diversity
DH: Doubled haploid
DSR: Diploid seed rate
E_RS%_: Relative efficiency of the response to selection selection
E_VG_: Estimation of the potential genetic variance
FDR: False discovery rate
HIR: Haploid induction rate
ISR: Inhibition seed rate
MAF: Minor allele frequency
N_e_: Inbreeding effective population size
PIC: Polymorphic information content
R*1-nj*: R*1-navajo* marker
RS: Response to selection
SNP: Single nucleotide polymorphism

## References

[1] Melchinger AE, Schipprack W, Friedrich Utz H, Mirdita V. In Vivo Haploid Induction in Maize: Identification of Haploid Seeds by Their Oil Content. Crop Science. 2014;54: 1497 10.2135/cropsci2013.12.0851

[2] Melchinger AE, Longin CF, Utz HF, Reif JC. Hybrid maize breeding with doubled haploid lines: quantitative genetic and selection theory for optimum allocation of resources. 2005; 14.

[3] Smith JSC, Hussain T, Jones ES, Graham G, Podlich D, Wall S, et al Use of doubled haploids in maize breeding: implications for intellectual property protection and genetic diversity in hybrid crops. Molecular Breeding. 2008;22: 51–59. 10.1007/s11032-007-9155-1

[4] Riggs TJ, Snape JW. Effects of linkage and interaction in a comparison of theoretical populations derived by diploidized haploid and single seed descent methods. Theoretical and Applied Genetics. 1977;49: 111–115. 10.1007/BF00281708 24407167

[5] Jannink J-L, Abadie TE. Inbreeding Method Effects on Genetic Mean, Variance, and Structure of Recurrent Selection Populations. Crop Science. 1999;39: 988 10.2135/cropsci1999.0011183X003900040006x

[6] Bernardo R. Should maize doubled haploids be induced among F1 or F2 plants? Theoretical and Applied Genetics. 2009;119: 255–262. 10.1007/s00122-009-1034-1 19396574

[7] Sleper JA, Bernardo R. Recombination and genetic variance among maize doubled haploids induced from F1 and F2 plants. Theoretical and Applied Genetics. 2016;129: 2429–2436. 10.1007/s00122-016-2781-4 27637886

[8] Nanda DK, Chase SS. An Embryo Marker for Detecting Monoploids Of Maize (Zea Mays L.)1. Crop Science. 1966;6: 213 10.2135/cropsci1966.0011183X000600020036x

[9] Melchinger AE, Schipprack W, Würschum T, Chen S, Technow F. Rapid and accurate identification of in vivo-induced haploid seeds based on oil content in maize. Scientific Reports. 2013;3 10.1038/srep02129 23820577 PMC3699789

[10] de Couto EGO, Davide LMC, de Bustamante FO, Pinho RGV, Silva TN. Identification of haploid maize by flow cytometry, morphological and molecular markers. Ciênc agrotec. 2013;37: 25–31. 10.1590/S1413-70542013000100003

[11] Rotarenco VA, Dicu G, State D, Fuia SRV. New inducers of maternal haploids in maize. 2010;84: 7.

[12] Chaikam V, Martinez L, Melchinger AE, Schipprack W, Boddupalli PM. Development and Validation of Red Root Marker-Based Haploid Inducers in Maize. Crop Science. 2016;56: 1678 10.2135/cropsci2015.10.0653

[13] Choe E, Carbonero CH, Mulvaney K, Rayburn AL, Mumm RH. Improving in vivo maize doubled haploid production efficiency through early detection of false positives: Improving maize doubled haploid production efficiency. Plant Breeding. 2012;131: 399–401. 10.1111/j.1439-0523.2012.01962.x

[14] Molenaar WS, Oliveira Couto EG, Piepho H, Melchinger AE. Early diagnosis of ploidy status in doubled haploid production of maize by stomata length and flow cytometry measurements. Plant Breeding. 2019;138: 266–276. 10.1111/pbr.12694

[15] Prigge V, Sánchez C, Dhillon BS, Schipprack W, Araus JL, Bänziger M, et al Doubled Haploids in Tropical Maize: I. Effects of Inducers and Source Germplasm on in vivo Haploid Induction Rates. Crop Science. 2011;51: 1498 10.2135/cropsci2010.10.0568

[16] Chaikam V, Nair SK, Babu R, Martinez L, Tejomurtula J, Boddupalli PM. Analysis of effectiveness of R1-nj anthocyanin marker for in vivo haploid identification in maize and molecular markers for predicting the inhibition of R1-nj expression. Theoretical and Applied Genetics. 2015;128: 159–171. 10.1007/s00122-014-2419-3 25385333

[17] Mahuku G, Chaikam V, Prasanna BM. Doubled Haploid Technology in Maize Breeding: Theory and Practice [Internet]. Mexico, D.F.: CIMMYT: BM Prasanna, Vijay Chaikam, George Mahuku; 2012. http://hdl.handle.net/10883/1351

[18] de Andrade LRB, Fritsche Neto R, Granato ÍSC, Sant’Ana GC, Morais PPP, Borém A. Genetic Vulnerability and the Relationship of Commercial Germplasms of Maize in Brazil with the Nested Association Mapping Parents. Chen C, editor. PLOS ONE. 2016;11: e0163739 10.1371/journal.pone.0163739 27780247 PMC5079593

[19] Chaikam V, Lopez LA, Martinez L, Burgueño J, Boddupalli PM. Identification of in vivo induced maternal haploids in maize using seedling traits. Euphytica. 2017;213 10.1007/s10681-017-1968-3 PMC7734196 33408421

[20] Geiger HH, Röber F DS. Vorträge für Pflanzenzüchtung. Methodology and genetics of in vivo haploid induction in maize. 1997 38: 203–244.

[21] Chase SS. Monoploids and Diploids of Maize: A Comparison of Genotypic Equivalents. American Journal of Botany. 1964;51: 928 10.2307/2440242

[22] Melchinger AE, Molenaar WS, Mirdita V, Schipprack W. Colchicine Alternatives for Chromosome Doubling in Maize Haploids for Doubled-Haploid Production. Crop Science. 2016;56: 559 10.2135/cropsci2015.06.0383

[23] Eder J, Chalyk S. In vivo haploid induction in maize. Theoretical and Applied Genetics. 2002;104: 703–708. 10.1007/s00122-001-0773-4 12582677

[24] Granato ISC, Galli G, de Oliveira Couto EG, e Souza MB, Mendonça LF, Fritsche-Neto R. snpReady: a tool to assist breeders in genomic analysis. Molecular Breeding. 2018;38 10.1007/s11032-018-0844-8

[25] Tateno Y, Nei M, Tajima F. Accuracy of estimated phylogenetic trees from molecular data: I. Distantly Related Species. Journal of Molecular Evolution. 1982;18: 387–404. 10.1007/bf01840887 7175956

[26] Stacklies W, Redestig H, Scholz M, Walther D, Selbig J. pcaMethods a bioconductor package providing PCA methods for incomplete data. Bioinformatics. 2007;23: 1164–1167. 10.1093/bioinformatics/btm069 17344241

[27] Yang J, Benyamin B, McEvoy BP, Gordon S, Henders AK, Nyholt DR, et al Common SNPs explain a large proportion of the heritability for human height. Nature Genetics. 2010;42: 565–569. 10.1038/ng.608 20562875 PMC3232052

[28] Stinard PS, Sachs MM. The Identification and Characterization of Two Dominant r1 Haplotype-Specific Inhibitors of Aleurone Color in Zea mays. Journal of Heredity. 2002;93: 421–428. 10.1093/jhered/93.6.421 12642642

[29] Hoisington DA, Nueffer MG, Coe EH Jr. The genetics of corn In: Corn and corn improvement. Madison, Wisconsin: Sprague G.F. and Dudley J.W. (eds.); 1988.

[30] Xu X, Li L, Dong X, Jin W, Melchinger AE, Chen S. Gametophytic and zygotic selection leads to segregation distortion through in vivo induction of a maternal haploid in maize. Journal of Experimental Botany. 2013;64: 1083–1096. 10.1093/jxb/ers393 23349137 PMC3580820

