# Supplementary material for: Effect of F1 and F2 generations on genetic variability and working steps of doubled haploid production in maize
Source: PLoS One. 2019 Nov 11;14(11):e0224631. doi: 10.1371/journal.pone.0224631 (PMC6844450; doi:10.1371/journal.pone.0224631)
Supplement: S1 Table — Generation (Fn), number of individuals (N°), inbreeding effective population size (Ne), estimation of the potential genetic variance (EVG), Nei’s genetic diversity (DG), polymorphic information content (PIC), minor allele frequency (MAF); coefficient of inbreeding (Fi). In parentheses are the maximum and minimum values. (DOCX) [file pone.0224631.s001.docx]

**Supporting Information**

**S1 Table**. Estimation of population parameters of DH lines obtained from each evaluated population: Generation (F_n_), number of individuals (Nº), inbreeding effective population size (N_e_), estimation of the potential genetic variance (E_VG_), Nei’s genetic diversity (D_G_), polymorphic information content (PIC), minor allele frequency (MAF); coefficient of inbreeding (F_i_). In parentheses are the maximum and minimum values

| **Germplasm sources** | **F_n_** | **Nº** | **N_e_** | **E_VG_** | **D_G_** | **PIC** | **MAF** | **F_i_** |  |
| --- | --- | --- | --- | --- | --- | --- | --- | --- | --- |
| DKB390 | F1 | 17 | 8.53 | 1955.7 | 0.25 (0.00 - 0.50) | 0.20 (0.00 - 0.37) | 0.19 (0.00 - 0.50) | 1.00 (0.99 - 1.00) |  |
| DKB390 | F2 | 13 | 6.53 | 1972.51 | 0.25 (0.00 - 0.50) | 0.20 (0.00 - 0.38) | 0.19 (0.00 - 0.50) | 1.00 (0.99 - 1.00) |  |
| 2B587PW | F1 | 20 | 10.02 | 1887.41 | 0.24 (0.00 - 0.50) | 0.19 (0.00 - 0.38) | 0.19 (0.00 - 0.50) | 1.00 (0.99 - 1.00) |  |
| 2B587PW | F2 | 15 | 7.53 | 1826.95 | 0.23 (0.00 - 0.50) | 0.19 (0.00 - 0.38) | 0.17 (0.00 - 0.50) | 1.00 (0.99 - 1.00) |  |
| BM820 | F1 | 19 | 9.50 | 1832.15 | 0.23 (0.00 - 0.50) | 0.18 (0.00 - 0.37) | 0.18 (0.00 - 0.50) | 1.00 |  |
| BM820 | F2 | 14 | 7.01 | 1651.19 | 0.21 (0.00 - 0.50) | 0.16 (0.00 - 0.38) | 0.16 (0.00 - 0.50) | 1.00 (0.99 - 1.00) |  |
| STATUS VIPTERA | F1 | 23 | 11.52 | 1839.59 | 0.23 (0.00 - 0.50) | 0.18 (0.00 - 0.38) | 0.19 (0.00 - 0.50) | 1.00 (0.99 - 1.00) |  |
| STATUS VIPTERA | F2 | 16 | 8.01 | 1735.8 | 0.21 (0.00 - 0.50) | 0.17 (0.00 - 0.38) | 0.17 (0.00 - 0.50) | 1.00 (0.99 - 1.00) |  |
| 30F53H | F1 | 16 | 8.03 | 2081.09 | 0.26 (0.00 - 0.50) | 0.21 (0.00 - 0.38) | 0.20 (0.00 - 0.50) | 1.00 (0.99 - 1.00) |  |
| 30F53H | F2 | 20 | 10.07 | 2036.02 | 0.26 (0.00 - 0.50) | 0.21 (0.00 - 0.38) | 0.19 (0.00 - 0.50) | 0.99 (0.99 - 1.00) |  |
